# Moderation effects of serotype on dengue severity across pregnancy status in Mexico

**DOI:** 10.1186/s12879-023-08051-z

**Published:** 2023-03-10

**Authors:** Esther Annan, Uyen-Sa D. T. Nguyen, Jesús Treviño, Wan Fairos Wan Yaacob, Sherry Mangla, Ashok Kumar Pathak, Rajesh Nandy, Ubydul Haque

**Affiliations:** 1 Department of Biostatistics & Epidemiology, University of North Texas Health Science Center, Fort Worth, TX USA; 2 Center for Health and Wellbeing, School of Public and International Affairs, Princeton University, Princeton, NJ USA; 3 Department of Urban Affairs at the School of Architecture, Universidad Autónoma de Nuevo León, 66455 San Nicolás de los Garza, Nuevo Léon México; 4 Mathematical Sciences Studies, College of Computing, Informatics and Media, Universiti Teknologi MARA Cawangan Kelantan, Lembah Sireh, Kampus Kota Bharu, 15150 Kota Bharu, Kelantan Malaysia; 5 Institute for Big Data Analytics and Artificial Intelligence (IBDAAI), Universiti Teknologi MARA, Kompleks Al- Hawarizmi, 40450 Shah Alam, Selangor Malaysia; 6 International Institute for Population Sciences, Mumbai, Maharashtra 400088 India; 7 Department of Mathematics and Statistics, Central University of Punjab, Bathinda, Punjab 151401 India; 8 Rutgers Global Health Institute, New Brunswick, NJ USA; 9 Department of Biostatistics and Epidemiology, School of Public Health, Rutgers University, Piscataway, NJ USA

**Keywords:** Mexico, Dengue, Severity, Severe, Pregnancy, Serotype, Women, Reproductive age

## Abstract

**Background:**

Pregnancy increases a woman’s risk of severe dengue. To the best of our knowledge, the moderation effect of the dengue serotype among pregnant women has not been studied in Mexico. This study explores how pregnancy interacted with the dengue serotype from 2012 to 2020 in Mexico.

**Method:**

Information from 2469 notifying health units in Mexican municipalities was used for this cross-sectional analysis. Multiple logistic regression with interaction effects was chosen as the final model and sensitivity analysis was done to assess potential exposure misclassification of pregnancy status.

**Results:**

Pregnant women were found to have higher odds of severe dengue [1.50 (95% CI 1.41, 1.59)]. The odds of dengue severity varied for pregnant women with DENV-1 [1.45, (95% CI 1.21, 1.74)], DENV-2 [1.33, (95% CI 1.18, 1.53)] and DENV-4 [3.78, (95% CI 1.14, 12.59)]. While the odds of severe dengue were generally higher for pregnant women compared with non-pregnant women with DENV-1 and DENV-2, the odds of disease severity were much higher for those infected with the DENV-4 serotype.

**Conclusion:**

The effect of pregnancy on severe dengue is moderated by the dengue serotype. Future studies on genetic diversification may potentially elucidate this serotype-specific effect among pregnant women in Mexico.

**Supplementary Information:**

The online version contains supplementary material available at 10.1186/s12879-023-08051-z.

## Introduction

In Mexico, it is estimated that 139,000 symptomatic dengue fever (DF) cases occur yearly on average, with an estimated yearly cost of $170 million and an average annual disease burden of 65 disability-adjusted life years (DALYs) per million population [1]. The overlap between DF symptoms and the physiological alterations seen among women during pregnancy may make the identification of warning signs difficult [2]. However, for identified cases, pregnancy increases the risk of hospitalization and the development of severe dengue [2].

Maternal mortality rates (MMR) vary across and within regions, in Mexico [3]. MMR is associated with factors like pregnancy-related hypertension, obstetric hemorrhage, quality of health care [4], and infections like DF [5]. Severe DF is associated with a high rate of fetal distress, intrauterine death, obstetric hemorrhage, preeclampsia and eclampsia, caesarian section deliveries, and death due to multiple organ failure days after delivery [5, 6]. Prior studies have also implicated DF in vertical transmission during late pregnancy, and implicated serotypes have been serotypes 1 and 2 (DENV-1 and DENV-2) [7]. DF-specific serotypes have been linked with severe outcomes of DF. Severer complications have mostly been associated with the DENV-2 serotype [8]. There is, however, a gap in literature portraying how the severity of DF in pregnancy is modified by the DF serotype in Mexico.

Other factors related to severe outcomes of DF are comorbidities. Adults with self-reported hypertension have 1.6 times the odds of developing dengue hemorrhagic fever (DHF) compared to non-hypertensives [9], while diabetes is associated with 2.75 times the odds of DHF [10]. Diabetes presents with a far worse prognosis in Mexico compared to high-income countries [11]. Approximately 20% of preventable deaths in Mexico are attributable to diabetes [12] and account for one-third of all mortality between the ages of 35 and 74 years [11]. Prior studies assert that the risk of dying among individuals hospitalized for dengue increases 11-fold when there are underlying comorbidities like diabetes [13]. A recent study done in Mexico, Brazil, and Colombia explored mortality associated with DF and found that comorbidities increase case fatality rates 3–17-fold [14].

This study aimed to explore (i) the moderation effects of DF serotype on pregnancy in causing severe DF. (ii) The spatial distribution of severe and non-severe dengue across pregnancy status in Mexico. Findings from this will inform policies regarding the management of DF, particularly among pregnant women.

## Method

### Data collection

The dataset used in the analysis was retrieved from Mexico’s Ministry of Health and contains non-identifiable health information collected from notifying health units across 2469 Mexican municipalities from 2012 to 2020. The total sample size was 94,832 women.

### Definition of variables

**Fig. 1:**
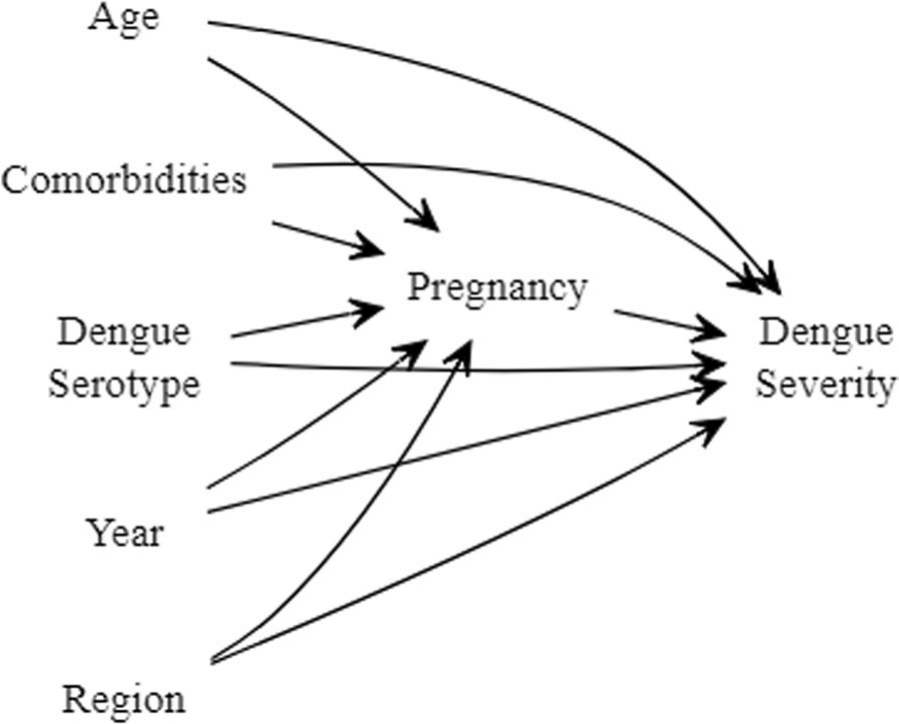
A directed acyclic graph of factors associated with pregnancy and dengue severity

Figure 1 shows a directed acyclic graph (DAG) of factors associated with pregnancy and dengue severity. Analysis was restricted to women of their reproductive age and defined by an age range of 15 to 49 years [15]. At each municipality clinic, women presenting with febrile illness characteristic of dengue fever were further tested for dengue antigens. An individual was defined as having dengue if she had a clinical diagnosis of DF and there was laboratory-confirmed evidence of non-structural protein (NS1) of DENV or a positive immunoglobulin M (IgM). DENV serotypes were determined based on polymerase chain reaction RT-qPCR results [16]. An individual with confirmed dengue had either DENV-1, DENV-2, DENV-3, or DENV-4 serotype. An individual with dengue was reported as having non-severe dengue, severe dengue, dengue without warning signs, dengue with warning signs, or ‘other’. Individuals with no dengue classification or ‘other’ classification were excluded from the analysis. The World Health Organization’s revised 2009 classification of DF emphasizes the inclusion of warning signs as a diagnostic criterion for probable and potentially severe dengue [17]. However, this classification requires laboratory-confirmed results to prevent inflation of the number of severe dengue cases. Because our dataset contained both clinical and laboratory-confirmed diagnoses, severe dengue was defined as individuals with severe dengue or having dengue with warning signs, while non-severe dengue was defined as individuals ‘having non-severe dengue or dengue without warning signs’. A woman was identified as pregnant or not pregnant based on pregnancy status classification retrieved from the dataset. Region was categorized as Center, Center West, Northeast, Northwest, and Southeast. Classification of the region has been defined elsewhere [18]. Hypertension and Diabetes were binary variables with ‘1’ indicating the presence of disease and ‘0’ indicating the absence of disease.

### Analysis

i) Model selection exploring the moderation effect of DENV serotype on dengue severity across pregnancy status

A multicollinearity test was performed and tolerance for hypertension (0.93) and diabetes (0.93) showed no multicollinearity between them. Similarly, there was no multicollinearity observed among the other covariates. Year and region were considered as potential random effects and were evaluated in a two-level hierarchical model. The Wald tests for random effects for the region (p = 0.049) and year (p = 0.032) were both significant. A comparison between random and fixed effects model using the Bayesian information criteria (BIC) revealed that the random-effects model performed better. Hence, both region and year were retained in the model as random effects. Based on the hierarchical model (1), a multiple logistic regression was performed with severe or non-severe DF as the outcome variable and pregnancy as the exposure variable.

1 $$y=X*\beta +Z*u+{\varvec{\varepsilon}}$$

where X a matrix (N *p) with p predictor variables and Z is a matrix (N*q) for q random effects [19].

Two-way interactions were explored, and multiple interactions were found to be statistically significant and retained. Other covariates in the model included dengue serotype, diabetes, hypertension, and age. The final model was chosen by comparing AIC and BIC between models. The AUC for the predicted probabilities of the final model was 0.7156 (Additional file 1: Fig. S1).

### Sensitivity analysis

The dataset shows the results of individuals who were tested for pregnancy, diabetes, and hypertension. However, individuals without a test could potentially have been misclassified as ‘negative’. To quantitatively assess for this kind of systematic error, a sensitivity analysis is recommended [20]. Using a misclassification spreadsheet [21], results from the regression analysis were explored for exposure bias. The misclassification spreadsheet provides adjusted bias data based on observed data on pregnant women stratified by dengue severity. As suggested for best practices [22], pairs of sensitivity and specificity were explored to study exposure misclassification.

ii) Spatial analysis

The sums of severe and non-severe dengue cases were calculated by pregnancy status. The spatial distribution of severe and non-severe dengue was visualized and compared across pregnancy status using ArcGIS. To measure spatial autocorrelation, Moran’s Index (I) was calculated for both pregnant and non-pregnant women with severe dengue. Moran’s I for the attributes pregnant (0.010, p = 0.13) and non-pregnant women (0.003, p = 0.54) were both not statistically significant, and the spatial distributions of these two attributes were random.

### Coding and environment

Data preprocessing, analysis, and generation of figures were done using SAS (version 9.4, SAS Institute Inc., Cary, NC, USA), R (version 4.1.2, The R Foundation, Vienna, Austria), and STATA/SE (Stata Corp LLC, College Station, TX, USA). All codes can be found in the Additional file.

### Sample size determination

A study conducted in Brazil found that 4 out of 707 (0.006) pregnant women and 19 out of 15,576 (0.001) non-pregnant had severe dengue [2]. To determine such an association with a power of 0.80, at an alpha of 0.05, a total sample size of 4547 was needed. The sample size was determined using the G*Power 3.1.9.4 software. Our study was comprised of 94,832 women of their reproductive age, of which 4943 were pregnant (5.21%) and 25,018 (26.38%) of them had severe dengue.

### Ethical approval

This study was reviewed and approved by the ethics and research committee of the Universidad Autónoma de Nuevo León and “North Texas Regional Institutional Review Board” as an exempt category (reference # 2021-035). All methods were performed in accordance with the DECLARATION OF HELSINKI guidelines for reporting observational studies. The need for informed consent was waived by the North Texas Regional IRB because the data analyzed was aggregated, de-identified and delinked, and therefore, obtaining informed consent was not applicable.

## Results

Table 1 shows the distribution of the sample of women from 2012 to 2020. The average age was 29 years old, with most women living in the Southeast region (50.12%) of Mexico. Across regions, DENV-2 was the commonest serotype found among individuals with severe DF (Additional file 2: Fig. S2). Compared to the other four regions, pregnant women in the Northeast region had the highest proportion of DENV-2 serotype (Additional file 3: Fig. S3). The chi-square statistic for the differences observed across various regions for dengue severity was statistically significant (χ^2^ = 2782.29, p < 0.0001). Among pregnant women, 33.57% had severe dengue, compared to 25.23% of non-pregnant women (Additional file 6: Table S1). The difference observed between pregnancy status for dengue classification was statistically significant (χ^2^ = 187.12, p < 0.0001).

**Table 1:** Demographic characteristics by dengue classification

Parameter	Dengue classification, n (%)			
		Severe dengue	Non-severe dengue	All
Region**	Center	1968 (7.87%)	6490 (9.30%)	8458 (8.92%)
	Center-West	4805 (19.21%)	18580 (26.61%)	23385 (24.66%)
	North-East	2084 (8.33%)	9856 (14.12%)	11940 (12.59%)
	North-West	1817 (7.26%)	8059 (11.54%)	9876 (10.41%)
	South-East	14344 (57.33%)	26829 (38.43%)	41173 (43.42%)
Pregnancy status**	Pregnant	1707 (6.82%)	3236 (4.64%)	4943 (5.21%)
	Not Pregnant	23311 (93.18%)	66578 (95.36%)	89889 (94.79%)
Age in years (SD)**		29.46 (± 9.87)	29.61 (± 9.75)	29.57 (± 9.78)
Year**	2012	6031 (24.11%)	11560 (16.56%)	17591 (18.55%)
	2013	6251 (24.99%)	15547 (22.27%)	21798 (22.99%)
	2014	2873 (11.48%)	8667 (12.41%)	11540 (12.17%)
	2015	1930 (7.71%)	8591 (12.31%)	10521 (11.09%)
	2016	9 (0.04%)	20 (0.03%)	29 (0.03%)
	2017	825 (3.30%)	4580 (6.56%)	5405 (5.70%)
	2018	1085 (4.34%)	3162 (4.53%)	4247 (4.48%)
	2019	4471 (17.87%)	10325 (14.79%)	14796 (15.60%)
	2020	1543 (6.17%)	7362 (10.55%)	8905 (9.39%)
Total (n)		25018 (26.38%)	69814 (73.62%)	94832 (100.00%)

^**^Chi-square or t-test performed for group differences had p < 0.001

Dengue severity has had both downward and upward trends from 2012 to 2020 (Additional file 4: Fig. S4). Among women with severe dengue, DENV-2 was the commonest variant, while DENV-1 was the commonest variant among those with non-severe dengue (Table 2). There was a similar distribution by pregnancy category; while most pregnant women had DENV-2, non-pregnant women mostly had the DENV-1 variant (Additional file 6: Table S1). DENV-2 was the commonest serotype in the Southeast region, while DENV-1 was the commonest serotype among women in the other regions of Mexico (Additional file 6: Table S2). The Southeast region had the highest proportion of severe dengue cases compared to other regions and this difference was statistically significant (p < 0.0001) (Additional file 6: Table S2).

**Table 2:** Proportions and p-value for different Chi-squared test results with 95% significance

Parameter	Dengue classification, n (%)			Chi-square p-value
		Non-severe dengue	Severe dengue	
Serotype	DENV-1	9825 (53.65%)	1887 (31.16%)	973 (< .0001)
	DENV-2	8145 (44.48%)	4081 (67.40%)	
	DENV-3	167 (0.91%)	63 (1.04%)	
	DENV-4	175(0.96%)	24 (0.40%)	
Hypertension	Hypertension	249 (0.36%)	465 (1.86%)	556 (< .0001)
	No Hypertension	69565 (99.64%)	24553 (98.14%)	
Diabetes	Diabetes	342 (0.49%)	566 (2.26%)	610 (< .0001)
	No Diabetes	69472 (99.51%)	24452 (97.74%)	
IgG	Positive	12655 (83.45%)	2754 (93.17%)	183 (< .0001)
	Negative	2509 (16.55%)	202 (6.83%)	
IgM	Positive	18388 (80.49%)	11594 (95.42%)	1441 (< .0001)
	Negative	4457 (19.51%)	556 (4.58%)	

**Fig. 2:**
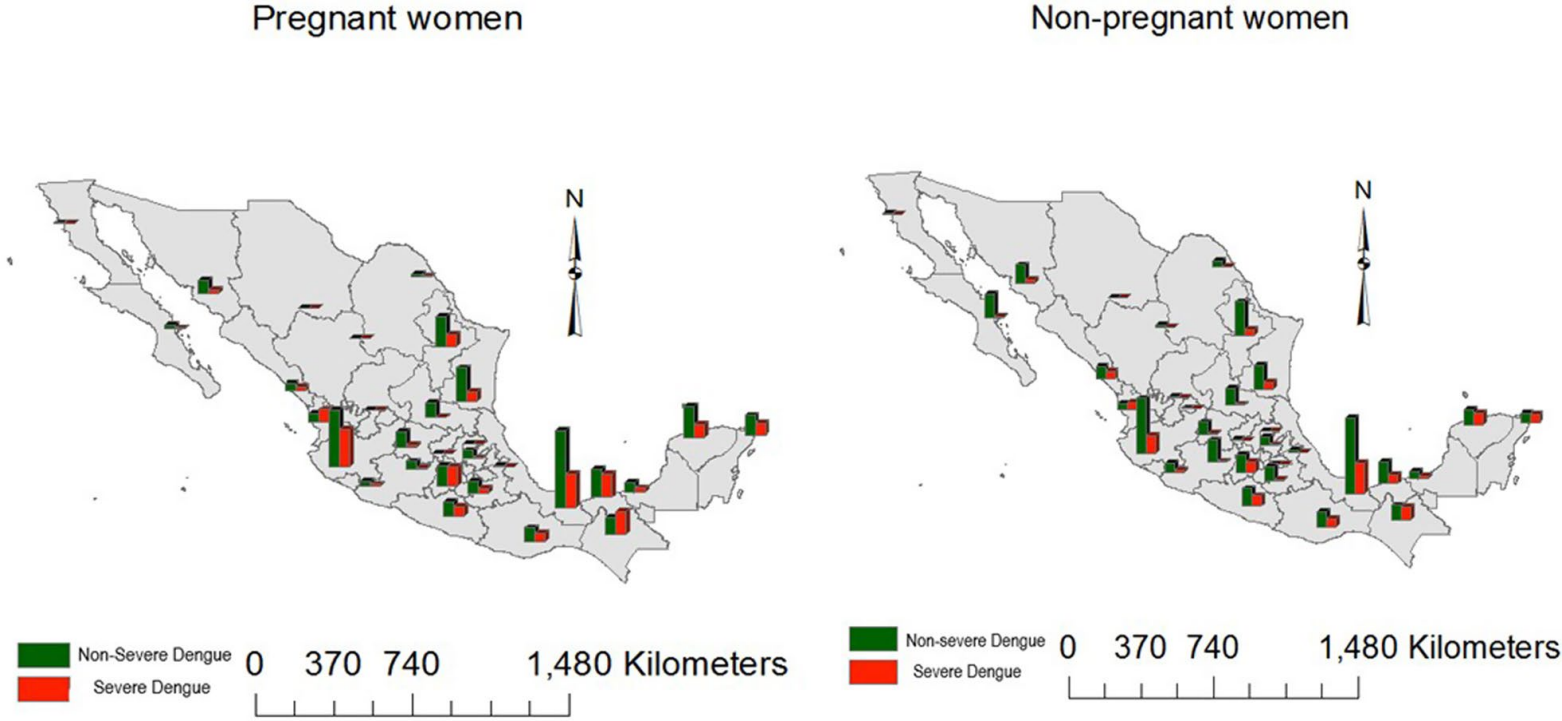
Dengue distribution among pregnant and non-pregnant women from 2012 to 2020

Additional file 5: Fig. S5 shows variations in severe dengue prevalence from 2012 to 2020 across Mexican states and pregnancy status. Both pregnancy strata showed a similar pattern of spread of severe dengue, although the number of cases in non-pregnant women was higher. A look at the proportions between severe and non-severe dengue for each pregnancy strata shows variations across states (Fig. 2). While most states recorded higher counts of non-severe dengue compared with severe dengue, Chiapas in the Southeast region, and Nayarit in the Center west region had a higher prevalence of severe dengue for pregnant women. This was contrasted with non-pregnant women who had similar proportions across severity strata in both Chiapas and Nayarit.

Unadjusted odds of a pregnant woman experiencing severe dengue were 1.5 times the odds of that of non-pregnant women. When adjusted for in a multiple logistic regression model, serotype moderated the effect of pregnancy. Respectively, among individuals with DENV-1, DENV-2, and DENV-4, pregnant women had 1.45, 1.35, and 3.78 times the odds, of severe dengue, compared to non-pregnant women after adjusting for other variables. Compared to those living in the Southeast region, individuals living in the Center, Center West, Northeast, and Northwest regions, had 0.38, 0.31, 0.37, and 0.66 times the odds of severe dengue (Table 3). Based on the random effects from yearly variations, on average, severe DF cases were higher in 2014 and lower in 2017 and 2020, compared to non-severe dengue (Additional file 6: Table S3). Similarly, severe DF cases were significantly higher in the Southeast and Center west regions. A one-unit increase in age was associated with 0.992 times lower odds of severe dengue after adjusting for other variables. All else equal, individuals diagnosed with diabetes had 2.6 times the odds, while those with hypertension had about 3.0 times the odds of severe dengue compared to those without diabetes and hypertension respectively. The variance seen among individuals with DENV-4 between pregnant and non-pregnant women is higher compared to the other serotypes. At a specificity of 99%, and sensitivity of 90% the adjusted OR for pregnancy was 1.63 (CI 1.52, 1.75) (Additional file 6: Table S4) compared to the unadjusted OR of 1.49 (CI 1.41, 1.59).

**Table 3:** Univariate and multivariate logistic regression showing predictors of the odds of dengue severity

Independent Variables	Univariate			Multivariate		
	OR	95% CI	*p-value	AOR	95% CI	p-value
Pregnant						
Yes, vs No	1.497	1.413, 1.587	0.0001	1.42	0.95, 2.11	< .0001
Age						
	0.997	0.996, 0.999	0.0003	0.99	0.99, 0.99	< .0001
Year						
	0.942	0.937, 0.947	0.0001	–	–	–
Serotype						
DENV-2 vs DENV-1	2.570	2.419, 2.731	0.0001	2.95	2.63, 3.30	< .0001
DENV-3 vs DENV-1	1.793	1.368, 2.350	0.0001	0.94	0.56, 1.56	0.0128
DENV-4 vs DENV-1	0.677	0.441,1.038	0.0737	0.59	0.32, 1.08	< .0001
Diabetes						
Yes, vs No	4.565	4.014, 5.193	0.0001	2.58	1.95, 3.40	< .0001
Hypertension						
Yes, vs No	5.365	4.620, 6.229	0.0001	2.97	2.14, 4.10	< .0001
Region						
Center vs Southeast	0.565	0.535, 0.597	0.0001	–	–	–
Center West vs Southeast	0.482	0.464, 0.500	0.0001	–	–	–
Northeast vs Southeast	0.396	0.376, 0.417	0.0001	–	–	–
Northwest vs Southeast	0.422	0.400, 0.446	0.0001	–	–	–
Pregnant vs non-pregnant						
DENV-1	–	–	–	1.45	1.2, 1.7	< .0001
DENV-2	–	–	–	1.33	1.2, 1.5	< .0001
DENV-3	–	–	–	0.55	0.2, 1.5	0.2438
DENV-4	–	–	–	3.78	1.1, 12.6	0.0305

^*^p-values are adjusted, using false discovery rate (FDR)

## Discussion

Our study found that the association between pregnancy and severe DF is moderated by the DENV serotype. The effect of DENV-4 in pregnant women may indicate effects of genetic diversification and the emergence of new serotype-specific genotypes [23, 24] and this warrants further investigation. Further, compared to other regions, the Southeast region had higher odds of severe DF. Dengue control programs and policies need to be expanded, using a multidisciplinary approach across Mexico.

Although a previous study found individuals with DENV-2 to have a lower risk of dengue hemorrhagic fever [25], the association between DENV-2 and higher risk of severe dengue, particularly when compared to DENV-1 [26], is consistent with most literature [26–29]. Similarly, DENV-2 and DENV-3 are more commonly associated with severe dengue compared to DENV-4 [26]. A recent study in Brazil, found that pregnant women had 1.92 times the odds of having DENV-4 serotype compared to non-pregnant women [2]. However, the authors concluded that the persistence of DENV-4 in a region and a higher number of cases in particular years may have explained the results found [2]. Our study’s finding of similar associations in Mexico may point to other potential mechanisms inherent in serotype-specific variations and/or their interaction with the pregnancy status. Another potential mechanism could involve the presence of DENV-4 as a heterologous infecting serotype. A high predominance of DENV-4 has previously been implicated in an outbreak in Jember, Indonesia, an area that did not frequently report DENV-4 but previously had outbreaks due to DENV-1, DENV-2, and DENV-3 serotypes [30].

Pregnant women are generally more at risk and predisposed to certain clinal conditions [31]. The risk of hospitalization as well as DF severity tends to be higher among pregnant women compared to non-pregnant women [2]. A higher proportion of IgG-positive serology compared to IgM in pregnant women might point to a higher risk of severe DF among those with secondary infection. While a host’s genetic background and immune status may influence disease presentation, it is suggested that certain viral structures may aid in replication in human target cells [32]. Differences among DENV serotypes may be attributed to genotype-specific (within serotypes) variances [25, 32]. One potential explanation is genetic diversity from clade replacements [33] which may be independent of pregnancy status. A clade replacement of a DENV serotype may be associated with a decrease or an increase in the prevalence of a heterologous DENV serotype [34]. Although the evidence does not support the transmission of antigenically aberrant strains, prior research suggests the displacement of DENV genotypes of less epidemiological significance by more virulent genotypes [32]. Genetic diversification and an emergence of a DENV-4 genotype-I have been found in a molecular analysis in Brazil and parts of tropical and subtropical America [23]. This may explain the moderation effect of DENV-4 serotype specifically among pregnant women compared to non-pregnant women. This is contrary to what is expected in the general population, where DENV-2 has been mostly associated with severer outcomes and DENV-4 has generally been associated with causing clinically mild diseases [25, 28]. It is also worth noting that although the effect of DENV-4 was statistically significant, the large confidence interval and smaller sample size may have influenced this finding. Hence, future prospective studies which may involve phylogenetic analysis or gene sequencing may further explore DENV-4 specific effects among pregnant women and the moderation effect of dengue serotypes in pregnancy.

The changing trend of increasing and decreasing cases of severe dengue may indicate a change in programs/policies associated with dengue fever eradication. The local health system mainly spearheads the charge toward dengue prevention programs, with minimal effort from other sectors like water and sanitation [35]. When comparing regions, women in the Southeast regions had higher odds of severe dengue compared to those in other regions. Central regions on average had lower proportions of severe dengue. This may be explained by the fact that cities like Mexico City in the Center region are free of endemic mosquito-borne viral diseases [36]. With a subtropical climate and high elevation, there is a lower occurrence of *Aedes *spp. in Central Mexico [36]. On the contrary, Pacific and Coastal regions tend to be at a higher risk of dengue [37]. For instance, states like Oaxaca in the pacific and southeastern region is one among the most affected states in Mexico, and the persistence of high dengue cases has been attributed to the presence of all four serotypes, favorable climate, and socioeconomic level of the population [38].

Our study had several limitations. Firstly, only a confirmed diagnosis of pregnancy status was reported in the dataset. Hence, to address potential misclassification bias, a sensitivity analysis was performed to assess the misclassification of pregnancy status. The analysis showed that at a sensitivity and specificity higher than 80% and 97% respectively, findings from our study were conservative. Another limitation was missing data for all variables. Particularly, prevalence estimates for pregnant women in Zacatecas and Chihuahua were missing across the period of study. However, our sample size of 94,832 women, satisfied the requirement for this study. Also, the cross-sectional nature of the study limits the inferential interpretation of results from the logistic regression model. The unavailability of information about vaccination status and behavioral factors that affect mosquito control also limits the study’s ability to control for these confounders. However, the spatial trend analysis provides additional reasons for further exploration in future studies. Furthermore, since pregnant women are more likely than other women to visit the clinic for antenatal care and be hospitalized, the likelihood of being diagnosed with dengue might be higher than non-pregnant women. However, this assumes a high antenatal care uptake and secondly, it also assumes that serological tests are performed routinely for pregnant women. Serological tests are performed upon clinical diagnosis of dengue. Since most dengue cases are asymptomatic and might go unnoticed, an acute presentation with a febrile illness among women is likely to present to the clinic regardless of pregnancy status. Lastly, restriction of the data to only women with serotype data means the likely exclusion of more people with non-severe dengue. However, the proportions of missingness among those with severe (76%) and non-severe (74%) dengue were similar. I higher proportion of missingness among non-pregnant women (75%) compared to pregnant women (60%), however, this supports the theory that more frequent access to healthcare may influence more diagnoses among pregnant women. Future studies may prospectively collect serotype data to ensure further limitation of potential bias.’ has been added to the limitations.

## Conclusion

Pregnancy increases a woman’s risk of severe dengue. However, this may be modified by the DENV-specific serotype. Of note is the DENV-4 serotype, which is otherwise the least severe serotype in the general population. Across Mexican regions, the southeast region had the highest number of severe dengue cases. Particularly, perinatal care in states like Chiapas and Nayarit may warrant further surveillance. This may especially be important for individuals with comorbid conditions like hypertension and diabetes and under the age of 24 years. An intersectoral approach is still needed across Mexico, particularly in the Southeast region to address the risk of DF severity. Further research is needed to fully understand the moderation effect of dengue serotype in pregnancy.

## Supplementary Information

**Additional file 1: Figure S1.** ROC Curve for Hierarchical logistic regression model.

**Additional file 2: Figure S2.** Distribution of dengue serotype by Dengue Severity and region.

**Additional file 3: Figure S3.** Distribution of dengue serotype by Pregnancy status and region.

**Additional file 4: Figure S4.** Trend of dengue fever among Mexican women in their reproductive age.

**Additional file 5: Figure S5.** Prevalence of severe dengue among pregnant and non-pregnant women from 2012 to 2020

**Additional file 6: Table S1.** Proportions of parameters by pregnancy status and p-value for different Chi-Squared Test results with 95% significance. **Table S2.** Proportions of parameters by region and p-value for different Chi-Squared Test results with 95% significance. **Table S3.** Random effects output from logistic regression. **Table S4.** Sensitivity Analysis for the effect of pregnancy on severe dengue.

## Data Availability

The dengue data used in this study are not publicly downloadable but can be requested at their original sites. Parties interested in data access should visit the Mexican Ministry of Health website (https://www.gob.mx/salud/en, E-mail: petitionscitizens@salud.gob.mx). The source data for the figures are available in Supplementary Data (Excel).
