# Supplementary figures and images for: Moderation effects of serotype on dengue severity across pregnancy status in Mexico
Source: BMC Infect Dis. 2023 Mar 10;23:147. doi: 10.1186/s12879-023-08051-z (PMC9999597; doi:10.1186/s12879-023-08051-z)

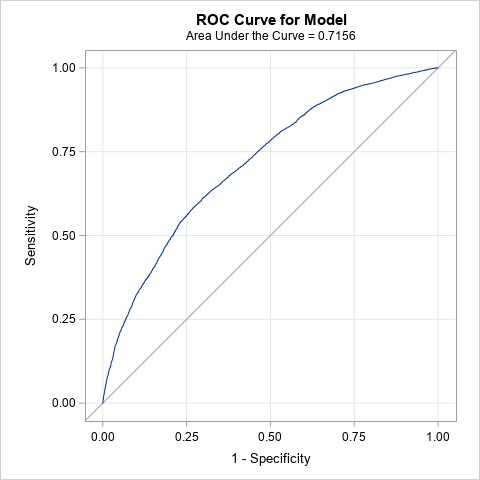

Supplement: Supplementary file 1 — Additional file 1: Figure S1. ROC Curve for Hierarchical logistic regression model. [file 12879_2023_8051_MOESM1_ESM.jpg]

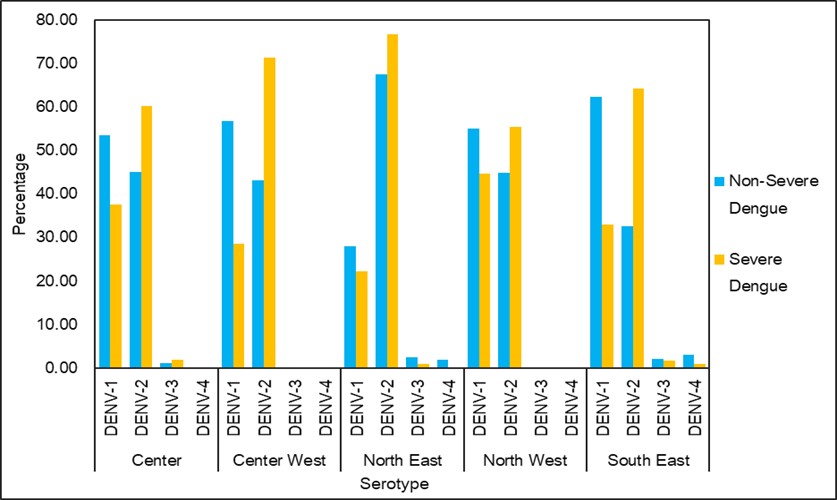

Supplement: Supplementary file 2 — Additional file 2: Figure S2. Distribution of dengue serotype by Dengue Severity and region. [file 12879_2023_8051_MOESM2_ESM.jpg]

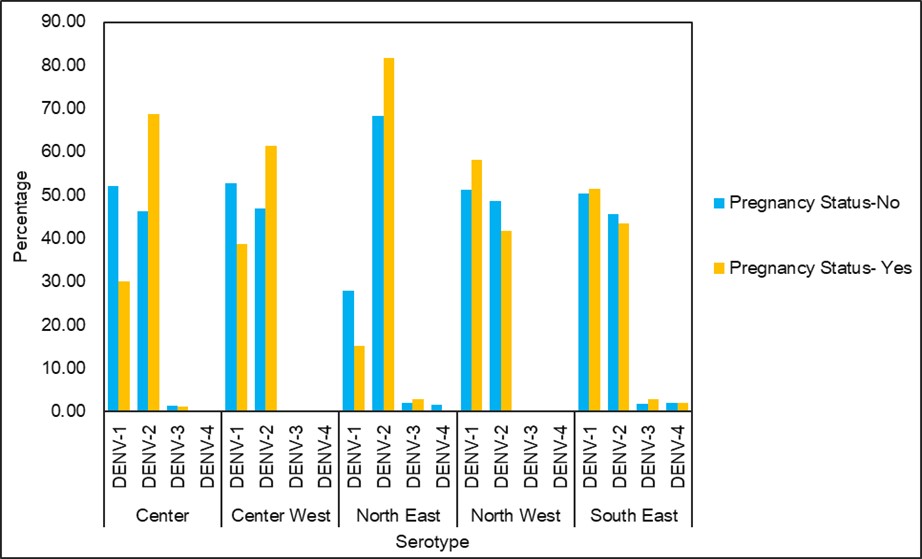

Supplement: Supplementary file 3 — Additional file 3: Figure S3. Distribution of dengue serotype by Pregnancy status and region. [file 12879_2023_8051_MOESM3_ESM.jpg]

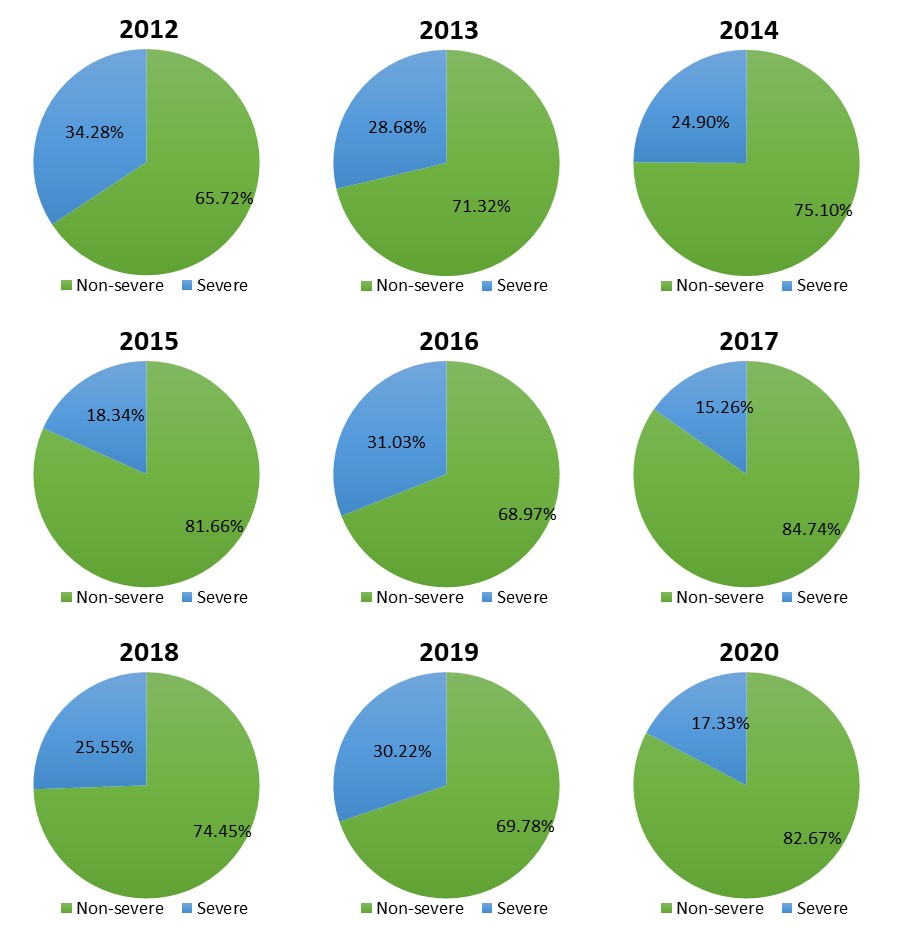

Supplement: Supplementary file 4 — Additional file 4: Figure S4. Trend of dengue fever among Mexican women in their reproductive age. [file 12879_2023_8051_MOESM4_ESM.jpg]

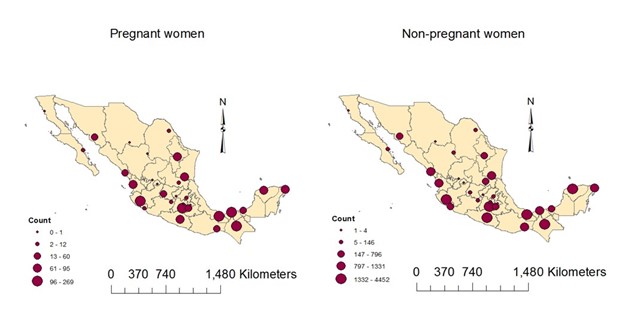

Supplement: Supplementary file 5 — Additional file 5: Figure S5. Prevalence of severe dengue among pregnant and non-pregnant women from 2012 to 2020 [file 12879_2023_8051_MOESM5_ESM.jpg]
