# Supplementary material for: Moderation effects of serotype on dengue severity across pregnancy status in Mexico
Source: BMC Infect Dis. 2023 Mar 10;23:147. doi: 10.1186/s12879-023-08051-z (PMC9999597; doi:10.1186/s12879-023-08051-z)
Supplement: Supplementary file 6 — Additional file 6: Table S1. Proportions of parameters by pregnancy status and p-value for different Chi-Squared Test results with 95% significance. Table S2. Proportions of parameters by region and p-value for different Chi-Squared Test results with 95% significance. Table S3. Random effects output from logistic regression. Table S4. Sensitivity Analysis for the effect of pregnancy on severe dengue. [file 12879_2023_8051_MOESM6_ESM.docx]

**Table S1: Proportions of parameters by pregnancy status and p-value for different Chi-Squared Test results with 95% significance**

| Parameter | | Pregnancy Status (%) | | |  |
| --- | --- | --- | --- | --- | --- |
|  |  | Non- pregnant | Pregnant | All | Chi-square p-value |
| Serotype | DENV-1 | 10949 (48.91%) | 763 (38.54%) | 11712 (48.07%) | <.0001 |
|  | DENV-2 | 11052 (49.37%) | 1174 (59.29%) | 12226 (50.17%) |  |
|  | DENV-3 | 201 (0.90%) | 29 (1.46%) | 230 (0.94%) |  |
|  | DENV-4 | 185 (0.83%) | 14 (0.71%) | 199 (0.82%) |  |
| Region | Center | 7989 (8.89%) | 469 (9.49%) | 8458 (8.92%) | <.0001 |
|  | Center- West | 22211 (24.71%) | 1174 (23.75%) | 23385 (24.66%) |  |
|  | North - East | 11305 (12.58%) | 635 (12.85%) | 11940 (12.59%) |  |
|  | North - West | 9640 (10.72%) | 236 (4.77%) | 9876 (10.41%) |  |
|  | South - East | 38744 (43.10%) | 2429 (49.14%) | 41173 (43.42%) |  |
| Hypertension | Negative | 89212 (99.25%) | 4906 (99.25%) | 94118 (99.25%) | 0.971 |
|  | Positive | 677 (0.75%) | 37 (0.75%) | 714 (0.75%) |  |
| Diabetes | Negative | 89020 (99.03%) | 4904 (99.21%) | 93924 (99.04%) | 0.212 |
|  | Positive | 869 (0.97%) | 39 (0.79%) | 908 (0.96%) |  |
| IgG | Negative | 2671 (15.42%) | 40 (4.99%) | 2711 (14.96%) | <.0001 |
|  | Positive | 14647 (84.58%) | 762 (95.01%) | 15409 (85.04%) |  |
| IgM | Negative | 4814 (14.20%) | 199 (18.37%) | 5013 (14.32%) | <.0001 |
|  | Positive | 29098 (85.80%) | 884 (81.63%) | 29982 (85.68%) |  |
| Dengue Classification | Non-Severe Dengue | 66578 (74.07%) | 3236 (65.47%) | 69814 (73.62%) | <.0001 |
|  | Severe | 23311 (25.93%) | 1707 (34.53%) | 25018 (26.38%) |  |
|  |  | **All (SD)** | **Pregnant (SD)** | **Not-Pregnant (SD)** | **t-test p-value** |
| Age in years | | 29.82(±9.88) | 24.91(±5.99) | 29.57(±9.78) | <.0001 |

**Table S2: Proportions of parameters by region and p-value for different Chi-Squared Test results with 95% significance**

| Parameter | |  | Region | | | | |  |
| --- | --- | --- | --- | --- | --- | --- | --- | --- |
|  |  | Center | Center-West | Northeast | Northwest | Southeast | All | Chi-square p-value |
| Serotype | |  |  |  |  |  |  |  |
|  | DENV-1 | 1193 (50.44%) | 6016 (51.78%) | 847 (26.78%) | 425 (51.64%) | 3231 (50.51%) | 11712 (48.07%) | <.001 |
|  | DENV-2 | 1137 (48.08%) | 5582 (48.04%) | 2201 (69.59%) | 398 (48.36%) | 2908 (45.46%) | 12226 (50.17%) |  |
|  | DENV-3 | 30 (1.27%) | 8 (0.07%) | 69 (2.18%) | 0 (0.00%) | 123 (1.92%) | 230 (0.94%) |  |
|  | DENV-4 | 5 (0.21%) | 13 (0.11%) | 46 (1.45%) | 0 (0.00%) | 135 (2.11%) | 199 (0.82%) |  |
| IgM | |  |  |  |  |  |  |  |
|  | Negative | 139 (5.57%) | 700 (14.95%) | 1505 (27.35%) | 303 (6.55%) | 2366 (13.37%) | 5013 (14.32%) | <.001 |
|  | Positive | 2358 (94.43%) | 3982 (85.05%) | 3997 (72.65%) | 4320 (93.45%) | 15325 (86.63%) | 29982 (85.68%) |  |
| IgG | |  |  |  |  |  |  |  |
|  | Positive | 37 (4.28%) | 247 (9.60%) | 2178 (46.50%) | 24 (2.15%) | 225 (2.53%) | 2711 (14.96%) | <.001 |
|  | Negative | 827 (95.72%) | 2327 (90.40%) | 2506 (53.50%) | 1091 (97.85%) | 8658 (97.47%) | 15409 (85.04%) |  |
| Dengue Classification | |  |  |  |  |  |  |  |
|  | Non-Severe | 6490 (76.73%) | 18580 (79.45%) | 9856 (82.55%) | 8059 (81.60%) | 26829 (65.16%) | 69814 (73.62%) | <.001 |
|  | Severe | 1968 (23.27%) | 4805 (20.55%) | 2084 (17.45%) | 1817 (18.40%) | 14344 (34.84%) | 25018 (26.38%) |  |

Table S3: Random effects output from logistic regression

| Parameter |  | Random Effects | | | | | | |
| --- | --- | --- | --- | --- | --- | --- | --- | --- |
|  |  | Intercept Estimate | | | Standard Error | P-Value | | |
| Region | Center | | -0.29 | 0.20 | | | 0.15 |  |
|  | Center West | | -0.48 | 0.20 | | | 0.01 |  |
|  | Northeast | | -0.31 | 0.20 | | | 0.12 |  |
|  | Northwest | | 0.27 | 0.20 | | | 0.19 |  |
|  | Southeast | | 0.68 | 0.20 | | | 0.0005 |  |
| Year |  | |  |  | | |  |  |
|  | 2012 | | -0.11 | 0.11 | | | 0.31 |  |
|  | 2013 | | 0.11 | 0.11 | | | 0.31 |  |
|  | 2014 | | 0.43 | 0.12 | | | 0.0006 |  |
|  | 2015 | | 0.19 | 0.13 | | | 0.14 |  |
|  | 2016 | | -0.02 | 0.29 | | | 0.93 |  |
|  | 2017 | | -0.26 | 0.12 | | | 0.03 |  |
|  | 2018 | | -0.08 | 0.11 | | | 0.50 |  |
|  | 2019 | | 0.18 | 0.11 | | | 0.09 |  |
|  | 2020 | | -0.50 | 0.11 | | | <.0001 |  |

Table S4: Sensitivity Analysis for the effect of pregnancy on severe dengue

| Sensitivity | Specificity | OR | SE |
| --- | --- | --- | --- |
| 80 | 99 | 1.634 | 0.036 |
| 85 | 99 | 1.631 | 0.036 |
| 90 | 99 | 1.628 | 0.036 |
| 80 | 98 | 1.854 | 0.045 |
| 85 | 98 | 1.850 | 0.045 |
| 90 | 98 | 1.847 | 0.044 |
| 80 | 97 | 2.326 | 0.062 |
| 85 | 97 | 2.322 | 0.062 |
| 90 | 97 | 2.318 | 0.061 |
